# Decreasing HMGB1 levels improves outcome of *Pseudomonas aeruginosa* keratitis in mice

**Published:** 2016-07-18

**Authors:** Linda D. Hazlett, Sharon A. McClellan, Sandamali A. Ekanayaka

**Affiliations:** 1 Department of Anatomy and Cell Biology, Wayne State University, School of Medicine, 540 East Canfield Avenue, Detroit, MI 48201, USA; 2 Department of Ophthalmology, Wayne State University, School of Medicine, 540 East Canfield Avenue, Detroit, MI 48201, USA

**Keywords:** HMGB1, Pseudomonas aeruginosa, Keratitis, Experimental model, Mice

## Abstract

*Pseudomonas (P.) aeruginosa* is a Gram negative bacterium widely dispersed in the environment which can cause acute and chronic infections in humans. According to the Centers for Disease Control and Prevention (CDC), the overall incidence of *P. aeruginosa* infections in USA hospitals averages about 0.4% (4/1000 discharges), and the bacterium is the fourth most commonly-isolated nosocomial pathogen accounting for 10.1% of all hospital-acquired infections. *P. aeruginosa* keratitis is a severe infection of the eye, progresses rapidly and remains a leading cause of corneal ulcers worldwide. Use of contact lenses is the major risk factor in the USA, while in less industrialized countries, trauma from agricultural accidents are of importance. Animal models of bacterial keratitis are of value in the study of this disease and suggest potential alternative therapeutic targets that are needed urgently due to increasing antibiotic resistance. Recently we have shown success and improved disease outcome after down-regulation of one promising target, high mobility group box1 (HMGB1) using small interfering RNA (siRNA). Testing more clinically relevant approaches are underway to reduce HMGB1 levels in *P. aeruginosa* keratitis which may hold promise for its treatment.

## Text

HMGB1 was originally described as a DNA-binding protein that functions as a structural co-factor for proper somatic cell transcription regulation^1,2^. Structurally, it is a small 215 amino acid protein, belonging to the family of danger associated molecular patterns (DAMPs) which amplify inflammatory reactions^2,3^. It is released extracellularly by necrotic and damaged cells and is recognized by the innate immune system to initiate repair processes. Extracellularly, HMGB1 promotes dendritic cell maturation and is also a potent pro-inflammatory cytokine^1^, contributing to tissue pathogenesis and inflammation from a variety of causes^1^. Increasingly reports indicate that it is a successful therapeutic target in experimental models of *P. aeruginosa*-induced pneumonia associated with cystic fibrosis^1^, and in sepsis^3–5^, arthritis^6^ and other diseases^7^. The molecule is a ligand for receptor for advanced glycation endproducts (RAGE) and induces nuclear translocation of NF-κB in macrophages, dendritic cells and neutrophils. It is detectable in the sputum of cystic fibrosis patients^1^ and in the serum of septic patients, where increases are in parallel with poor prognosis^3^. Monocytes^8^, macrophages^8,9^, natural killer cells^10^ and dendritic cells^11^ secrete HMGB1 in response to a variety of stimuli, including pathogen associated molecular patterns (PAMPS) (e.g., lipopolysaccharide, LPS), enhancing both innate and adaptive immunity.

Because of global emergence of antibiotic resistant bacterial pathogens, development of alternative therapeutic targets to treat microbial infections is an urgent need^12,13^. In this regard, extracellular HMGB1 is an attractive candidate, as it is a late mediator of the inflammatory response with levels plateauing between 24 to 36 hours after infection^5^.

This provides a wide therapeutic window and suggests that it may be an optimum target for development clinically. In fact, recently, our laboratory reported the use of a small interfering RNA (siHMGB1) to knock down HMGB1 in a mouse model of *P. aeruginosa* keratitis^14^. This prophylactic treatment led to improved disease outcome along with reduction in pro-inflammatory cytokines, an increase in anti-inflammatory cytokines and reduced neutrophil infiltration. For this, HMGB1 was silenced using small interfering RNA, whereas controls were treated with a non-specific scrambled sequence small interfering RNA. Less disease was seen post-infection in siHMGB1 compared with control mice and was documented by reduced clinical scores and less corneal opacity. Real-time RT-PCR and ELISA confirmed HMGB1 knockdown and that it was significant over scrambled controls. RT-PCR analysis revealed reduced mRNA levels of IL-1β, MIP-2, TNF-α, TLR4, and RAGE, whereas mRNA levels of anti-inflammatory TLRs single Ig IL-1–related receptor (SIGIRR) and ST2 were increased significantly. HMGB1 knockdown also decreased IL-1β and MIP-2 proteins, reducing neutrophils in the infected cornea. mRNA and protein levels of CXCL12 and CXCR4, as well as mononuclear cells, were reduced significantly after HMGB1 knockdown. These data provided evidence that silencing HMGB1 promoted better resolution of *P. aeruginosa* keratitis by decreasing levels of pro-inflammatory mediators (decreasing neutrophil infiltration), increasing anti-inflammatory TLRs, reducing CXCL12 (preventing HMGB1/CXCL12 heterodimer formation), and signaling through CXCR4 and reducing monocyte/macrophage infiltration^14^. These data also provided proof of principle to further develop more clinically relevant means to reduce levels of HMGB1, as silencing or knockdown in a clinical setting would not be optimum due to shortcomings such as longevity of treatment efficacy and possibly toxicity issues^15,16^.

Treatment with another molecule, vasoactive intestinal peptide (VIP), an anti-inflammatory neuropeptide, which was found to down-regulate HMGB1^17^ expression and promote healing in a susceptible (cornea perforates) model of *Pseudomonas aeruginosa* keratitis appeared to be efficacious^17–19^. However, the use of VIP is also problematic, as it is associated with difficulty in delivery and other issues^20^. So despite encouraging data, we did not pursue use of the neuropeptide, but turned to other approaches to reduce HMGB1 levels. For example, antibody neutralization of HMGB1 also improved disease outcome of keratitis in *P. aeruginosa* infected susceptible mice^14^. Similar therapeutic interventions with anti-HMGB1 antibodies were shown as an effective strategy to ameliorate HMGB1 mediated amplification of inflammation in diverse other experimental animal models of acute and chronic diseases^21^. In murine models of lethal sepsis, administration of neutralizing HMGB1 antibodies, initiated at 24 hours after infection, improved the survival rate of mice, while decreasing the production of HMGB1 induced pro-inflammatory cytokines^22,23^.

Box A^1^ is one of the three main domains of HMGB1and functions as an HMGB1 antagonist. Similar to anti-HMGB1 antibodies, animal studies revealed that treatment with HMGB1 Box A peptide inhibited the pro-inflammatory cytokine effects of HMGB1 and improved disease outcome in many infectious and non-infectious diseases^21^. In mouse models of sepsis, Box A treatment improved disease outcome and increased the survival rate of mice^23^.

We also tested the effects of thrombomodulin in the keratitis model^24^. Thrombomodulin (TM) is a multidomain transmembrane glycoprotein present in diverse cell types. Thrombomodulin domain (TMD) 1 is lectin-like, interacting with Lewis Y antigen on LPS, and with HMGB1^25^. The role of TM has been studied in inflammatory diseases in the eye, including endotoxin-induced uveitis^26^ and results show that in these diseases, TM expression is observed in the corneal epithelium and in stromal cells. The distribution of TM is similar in the eye of humans^27^ and mice^28^, suggesting a potential for similarity of function in both species. Evidence suggests that the lectin-like domain of TM, by reduction of HMGB1, may sequester its adverse effects^29,30^. Similar to siHMGB1, recombinant TM (rTM, comprised of TM domains 1–4, Leu 17-Ser 517), treatment significantly lowered clinical scores in treated mice at 3 and 5 days after infection and modestly (1 log) decreased viable bacteria in the cornea. In addition, this treatment lowered mRNA levels for several pro-inflammatory molecules including NF-κβ, TLR4, and RAGE. It also provided a modest, yet significant upregulation in anti-inflammatory cytokines such as SIGIRR^31^ and ST2^32^, which have been previously shown to contribute to better disease outcome. However, this treatment did not reduce levels of HMGB1 (mRNA or protein). Since the rTM used did not contain a complete domain 1, we also selectively tested another peptide that contained the full domain 1 (rTMD1). Treatment with the latter failed to modulate disease severity, mRNA levels of HMGB1, pro- or anti-inflammatory molecules tested. These data suggest that TM is protective in bacterial keratitis, despite the lack of observable effects on HMGB1 levels and that an alternate mechanism is responsible for the outcome.

Other molecules are also being tested, and include glycyrrhizin (GLY)^33^ a natural anti-inflammatory and antiviral triterpene and a synthetic derivative of GLY, carbenoxolone^34^ (CBX), to reduce HMGB1. Both are small triterpenoid saponin molecules that directly bind HMGB1, do not interfere with its secondary structure and inhibit HMGB1-mediated mitogenic and chemotactic functions^33^. Both GLY and CBX have been used clinically to treat chronic hepatitis^35^, allergic conjunctivitis and blepharitis^36^ with no complications in patients. Preliminary experiments in mice using invasive (clinical isolate, KEI 1025) and cytotoxic (ATCC strain 19660) pseudomonas strains, revealed that treatment reduced HMGB1 levels, bacterial plate count, inflammatory consequences and disease outcome, when given prophylactically and, most importantly, when topical treatment was delayed for 6 hours after infection (Hazlett, unpublished data). Taken together, these pre-clinical anti-HMGB1 treatment strategies provide routes to develop therapeutics based upon reduction of HMGB1 levels, with the goal of using this strategy to clinically manage the adverse effects of HMGB1 in keratitis.
